# Warm hole in Pacific Arctic sea ice cover forced mid-latitude Northern Hemisphere cooling during winter 2017–18

**DOI:** 10.1038/s41598-019-41682-4

**Published:** 2019-04-03

**Authors:** Yoshihiro Tachibana, Kensuke K. Komatsu, Vladimir A. Alexeev, Lei Cai, Yuta Ando

**Affiliations:** 1 Faculty of Bioresources, Mie University, Tsu, Japan; 2 International Arctic Research Center, University of Alaska Fairbanks, Fairbanks, USA

## Abstract

In North America and Asia, extreme cold weather characterized the winter of 2017–18. At the same time, the Pacific, the Bering Sea, and the Atlantic Arctic regions experienced anomalously low sea ice extent in the early winter. The jet stream dividing cold Arctic air from warm air deviated from normal zonal patterns northward into the ice-free areas north of the Bering Strait. Large southward jet stream pathways formed over Asia and America, allowing cold air to spread into Asia and the southern areas of North America. We hypothesise that the late autumn Bering Strait sea-ice anomaly and Pacific atmospheric rivers were partially responsible for the cold winter. We used data analyses and numerical experiments to test this hypothesis. We propose a positive feedback mechanism between the sea ice anomaly and atmospheric river activity, with anomalous south winds toward the sea ice anomaly potentially leading to more warm water injected by the wind-driven current through the Bering Strait. Our findings suggest that Poleward propagation of the atmospheric rivers made upper air warm, leading to their upgliding, which further heated the overlying air, causing poleward jet meanders. As a part of this response the jet stream meandered southward over Asia and North America, resulting in cold intrusions. We speculate that the positive feedback mechanism observed during the 2017–18 winter could recur in future years when the sea-ice reduction in the Pacific Arctic interacts with enhanced atmospheric river activity.

## Introduction

The winter of 2017–18 was abnormally cold over Eastern Canada and East Asia. Likewise, both Korea and Japan also experienced record-breaking winter cold. Data from the Japanese Meteorological Agency reveal that the seasonal mean temperature in western Japan was the lowest recorded since 1985–86^1^. This cold winter cannot be accounted for by a single cold wave event, which typically do not last throughout the winter. The negative phase of the Arctic Oscillation (AO), which is equivalent to a weak jet stream, is a good measure for hemispheric cold weather^2^. In this case, the AO was persistently in its negative phase from mid-November through mid-February. A weak jet is usually accompanied by large meanders^3–5^, in the same way that a slow-flowing fluvial river meanders. Negative AO does not specify where the southward meander travels, but in this case, the southward meander was locked in Eastern Canada and East Asia. This persistent southward meander allowed cold air to spread southward. Understanding the reason for this large persistent meander, with regional focal areas, yields explanations for the extreme cold weather in 2017–18. La Niña is also known as a cause of extreme weather, but because La Niña occurs approximately every four years, and because it was weak in this particular season, La Niña^6^ alone does not explain the cold weather of 2017–18.

One other possible explanation for the cold winter of 2017–18 is the reduction in sea ice extent off the northern coast of Norway, specifically the Barents Sea ice extent has been extremely low during the last decade. This semi-permanent ice reduction tends to make Eurasia cold^7–9^. It has been shown that anomalous heat flux from an ice-free ocean excites large-scale atmospheric stationary waves that travel eastward over Eurasia, causing cold weather in East Asia^7^. Ice reduction in the Barents Sea is also known to strengthen the downward influence of the stratosphere^10–12^, making the phase of AO negative and resulting in cold mid-latitudes. However, influence of the Barents Sea ice reduction mechanism has not been widely accepted yet. Numerical simulations need many ensemble runs in order to arrive at a statistically significant level to show the influence of the Barents Sea ice^9,12^. This is because the variability of the jet meanders owing to atmospheric internal fluctuation is much larger than that owing to surface conditions such as sea ice^7,9^. Barents Sea ice was not exceptionally low in the 2017–18 winter compared with recent years. Since Barents Sea ice has been extremely low during the last ten years, it should have been an extremely cold winter every year, which was not the case. The downward influence of the stratosphere^10–12^ was also not predominant in the 2017–18 winter, as stratospheric sudden warming, which tends to make the tropospheric mid-latitudes cold, had not occurred until late winter. Direct explanations for this severe winter thus remain unclear. In addition to the anomalous upward heat flux from an ice-free ocean, horizontal moisture flux injecting into the Arctic, such as atmospheric rivers, makes the Arctic warm^13–17^. Although both the upward and horizontal fluxes may excite large-scale atmospheric stationary waves that resultantly make mid-latitudes cold, no previous studies have demonstrated the influence of the horizontal flux upon the mid-latitudes.

Here we propose a new candidate: The Arctic Ocean Pacific sector, including the Chukchi Sea and Bering Sea, which experienced the lowest sea ice coverage^18^ of recent decades, resulting in the cold 2017–18 winter. The present study demonstrates that the sea ice in the Pacific Arctic was a chief cause of the cold winter in mid latitudes.

We first describe the anomalous jet meander and anomalous sea-ice coverage in winter 2017–18 from the global-scale viewpoint. Next, we show that sea-ice retreat over the Pacific Arctic sector was responsible for the anomalous winter by using atmospheric numerical experiments. We show how this area modulated the overlying atmosphere as viewed from a local atmosphere-ice-ocean feedback system.

## Results

### Anomalous jet meander during winter 2017–18 drives cold mid-latitudes and warm Arctic

**Figure 1:**
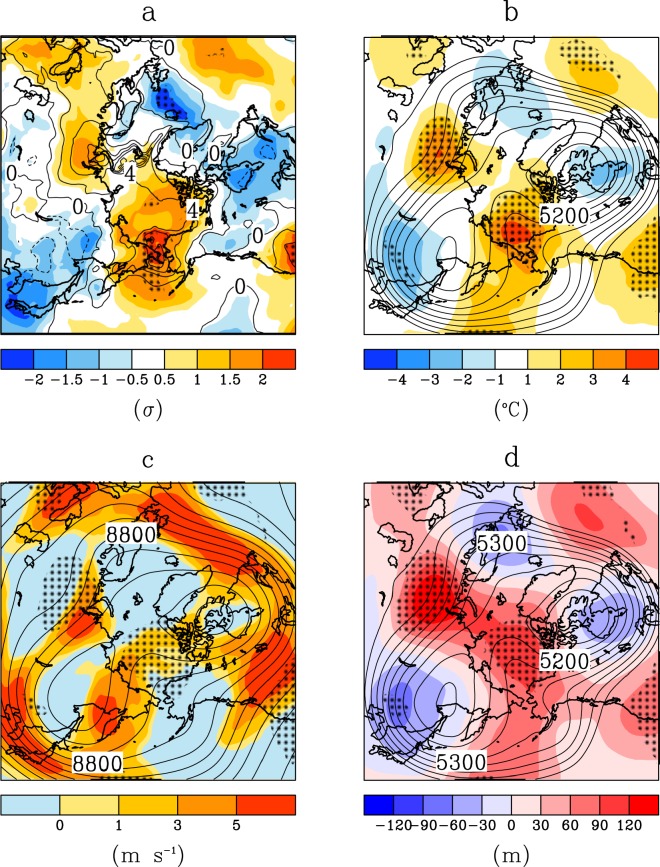
Anomalous jet meander, cold winter in Asia and America in winter 2017–18. (**a**) Three-month mean air temperature (contour) and standardized temperature anomaly (shaded) at 2 m in winter 2017–18 between 15 November 2017 and 15 February 2018. The anomalies were divided by their standard deviations and the data were linearly de-trended. The contour interval is 1 °C. Hatching areas recorded the highest or lowest temperature of the three-month means since 1981. (**b**) As in (**a**) but for geopotential height (contour) and temperature anomaly (shaded) at 500 hPa. The trend is not subtracted. The unit of height is meters, and its contour interval is 50 m. (**c**) As in (**b**) but for wind speed anomaly at 300 hPa and wind speed anomaly. (**d**) As in (**b**) but for geopotential height (contour) and its anomaly (shaded) at 500 hPa.

The 2017–18 winter was abnormally cold over wide areas in the mid latitudes in the Northern Hemisphere, in particular in Japan, Western Europe, and Eastern Canada; whereas near the Bering Strait, the winter was historically warm (Fig. 1a). These contrasting anomalous cold and warm areas vertically spread to the upper air (Fig. 1b). The upper air temperature in a portion of North-eastern Asia was also the coldest for all winters since 1981. A persistent southward jet stream meander covered East Eurasia and North America, allowing cold Arctic air to spread into these regions (see the contour for 5200 m in Fig. 1b, with jet flows along the geopotential height contours). Another significant factor was the jet’s intrusion from Alaska into the region near the North Pole for nearly the entire winter. The jet intrusion is also seen in wind speed anomalies in the upper troposphere (See circular belts meanders with reddish colour in Fig. 1c). The intrusion is a result of a historical maximum in the upper-air geopotential height and 500 hPa temperature over the Pacific Arctic sector (Fig. 1d). This extreme poleward jet intrusion in winter 2017–18 is therefore unprecedented.

### Historical minimum in November sea-ice cover over the Pacific sector of the Arctic

**Figure 2:**
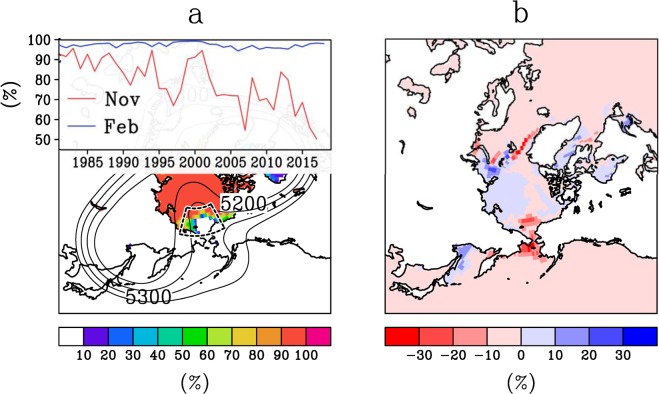
Sea-ice hole in winter 2017–18 and anomalous jet meander. (**a**) Map of monthly mean sea-ice concentrations in November 2017, and interannual variation of area-averaged monthly mean sea-ice concentration within a fan-shaped area in the Chukchi Sea for the months of November (red) and February (blue). The unit of height is meters, and its contour interval is 50 m. (**b**) Ice concentration deviation for winter 2017–18, from those of most recent five-year mean. Average period is from November to February.

Sea ice concentration in the Chukchi Sea was at an historical minimum in November, and closed at the end of December (Fig. 2a). Freeze-up was also anomalously late, and sea ice in the Chukchi and Bering Seas was still at its lowest concentration in January and February. Comparison with the most recent five-year mean shows the anomaly in this area (See Fig. 2b). The delayed freeze up, specifically, the phase change from liquid to solid water, would have provided the overlying air with heat, in delayed time, owing to the heat release of solidification. In addition, anomalous upward sensible heat and moisture flux from the ice-free ocean to the atmosphere assists atmospheric warming. Because the shape of the ice-free ocean appears as a hole in the larger ice cover, we refer to this sea-ice hole as a warm hole. Although sea ice formed over this area in January, the term ‘hole’ is used to refer to the anomalous ice condition.

Over the Bering Strait warm hole, the temperature was historically highest from the surface to the upper air (Fig. 1). The warm upper air allowed the jet stream to meander poleward, as the jet essentially marks the boundary between the cold polar air to its north and the warm air to its south. The contour of 5250 m in Fig. 2 shows that a portion of the jet detoured northward. To the south of the poleward jet meander, there was extremely warm air overlying a predominately ice-free ocean. In reaction to this anomalous northward meander, the jet also meandered in the opposite direction, to the south over East Eurasia and North America with southward cold intrusion^19,20^.

### Response of large-scale atmosphere to the warm hole in sea ice cover with numerical experiment

In order to identify the atmospheric response to the sea-ice reductions of individual areas, i.e., the warm hole in the Pacific sector of the Arctic, the Barents Sea, and other areas, atmospheric numerical experiments were performed. In order to see the difference we executed the following numerical experiments: one forced with largely ice-free boundary conditions observed in 2017–2018, and the other with sea ice corresponding to the high-ice period in 1983–1984.

**Figure 3:**
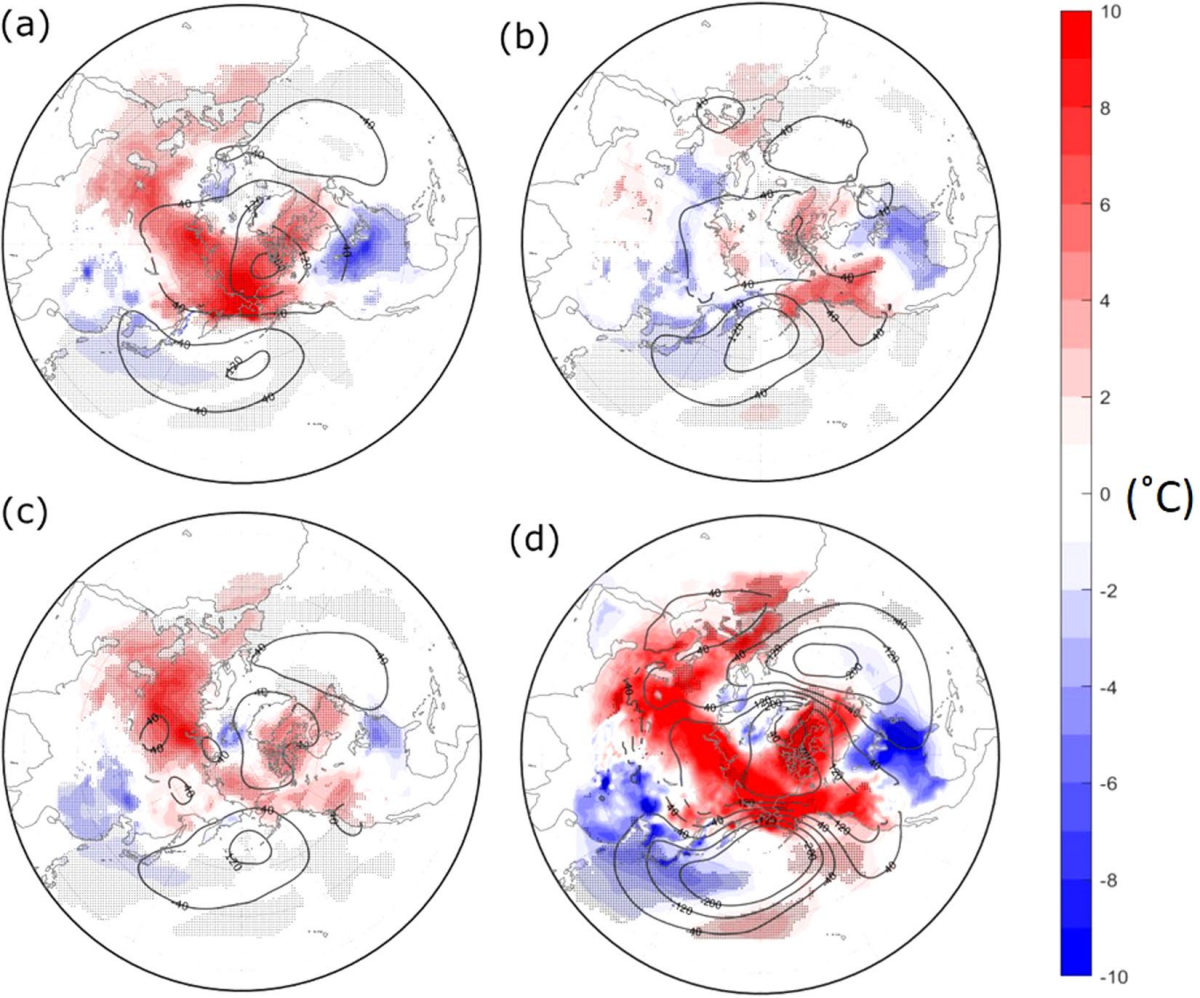
Atmospheric response under the 2017–18 sea-ice boundary condition by a numerical simulation in the lower troposphere. (**a**) Atmospheric deviation fields in February when sea-ice boundary condition over a specific region is set in 2017–18 from those of 1983–84. (**a**) Bering-Chukchi region, (**b**) Barents-Kara region, (**c**) Greenland-Hudson Bay region, (**d**) the sea ice of all the three regions is set in 2017–18. Contour and shaded are 850hP height and surface air temperature deviations respectively. The units are meter and °C. Hatched areas indicate the temperature deviation exceeds 95% confidence level by *t*-test.

**Figure 4:**
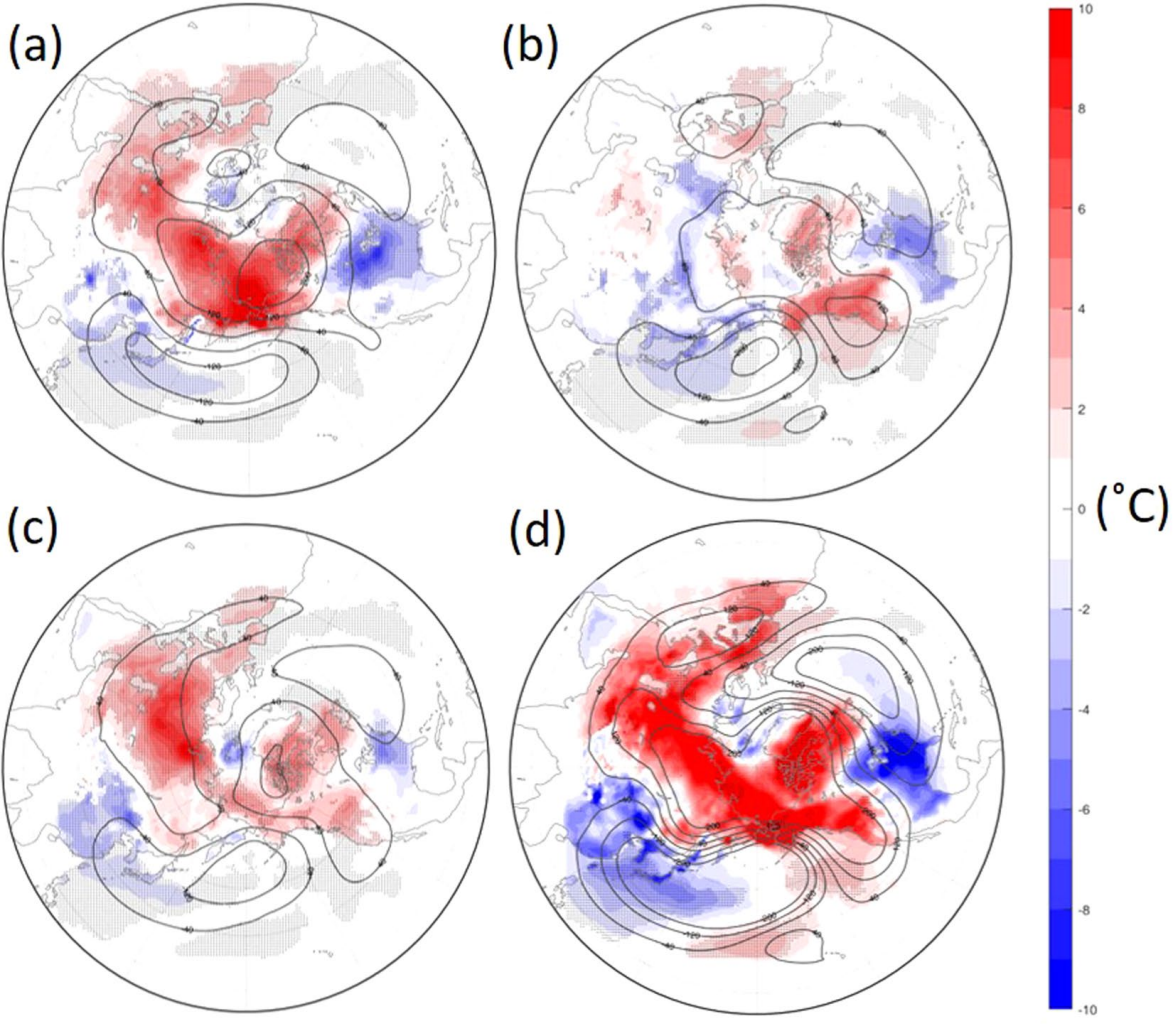
Atmospheric response under the 2017–18 sea-ice boundary condition by a numerical simulation in the middle troposphere. (**a**) Atmospheric deviation fields in February when sea-ice boundary condition over a specific region is set in 2017–18 from those of 1983–84. (**a**) Bering-Chukchi region, (**b**) Barents-Kara region, (**c**) Greenland-Hudson Bay region, (**d**) the sea ice of all the three regions is set in 2017–18. Contour and shaded are 500 hPa height and surface air temperature deviations respectively. The units are meter and °C. Hatched areas indicate the temperature deviation exceeds 95% confidence level by *t*-test.

An experiment under ice-free conditions in the Pacific side of the Arctic successfully simulated a cold East Asia and North America in the lower troposphere (Fig. 3a). This experiment successfully simulated a warm Arctic. The response in the middle troposphere is also similar to that of the lower troposphere (Fig. 4a), with positive 500 hPa height anomalies over the Arctic, and negative height anomalies over the North Pacific. The results are in agreement with the observed anomalies shown in Fig. 1, with little eastward phase shift.

Figure 3b,c show experiments under ice-free conditions in the Barents Sea and Greenland-Hudson Bay areas respectively. Interestingly, northeast Asia and North America are often cool when the ice-free region is over the Barents Sea or Greenland-Hudson Bay, as well as the Pacific sector. Although the response to the forcing of the Barents Sea also shows a cold East Asia, the horizontal anomaly patterns do not resemble the observations as well as that of the response to the warm hole (Figs 3 and 4). It should be noted that when all three regions are ice-free—which occurred during the 2017–18 winter— the atmospheric response pattern is more amplified than those of the simple sum of each run (Figs 3d and 4d), suggesting that some resonant effects may have occurred. Furthermore, a water vapour anomaly under ice-free conditions shows positive humidity anomalies over the Arctic region, though the statistical significance is not high (Fig. S1).

### Northward intrusion of moisture from the Pacific into the Arctic

**Figure 5:**
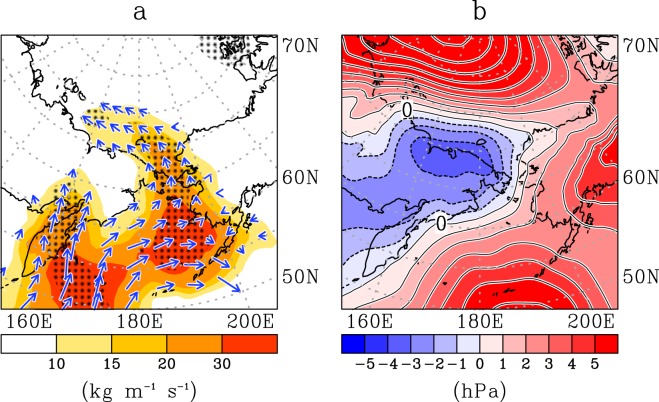
Anomalous poleward moisture flux and sea level pressure over the Pacific side of the Arctic Ocean and the Bering Sea in winter 2017–18. (**a**) Arrow shows the three-month mean vertically integrated moisture flux in winter 2017–18 between 15 November 2017 and 15 February 2018. Hatching areas exceeded two times as large as one standard deviation of the absolute value of the moisture flux. (**b**) Shaded shows the anomaly of moisture flux from its climatology. (Contour) Three-month mean sea level pressure (SLP) anomaly. Solid line shows positive SLP anomaly, and broken line shows negative SLP anomaly. Contour interval is 1 hPa.

The numerical experiments have shown that the warm hole in the Pacific sector played a role in forming a cold Asia and warm Arctic from the view point of large-scale atmospheric circulation. We focus on the area of the warm hole in order to understand how the warm hole promotes the jet meander. Figure 5a shows the three-month mean vertically integrated moisture flux in winter 2017–18. An anomalously large northward moisture flux penetrated into the Chukchi Sea from the North Pacific in association with cyclones centred to the west (Fig. 5b).

**Figure 6:**
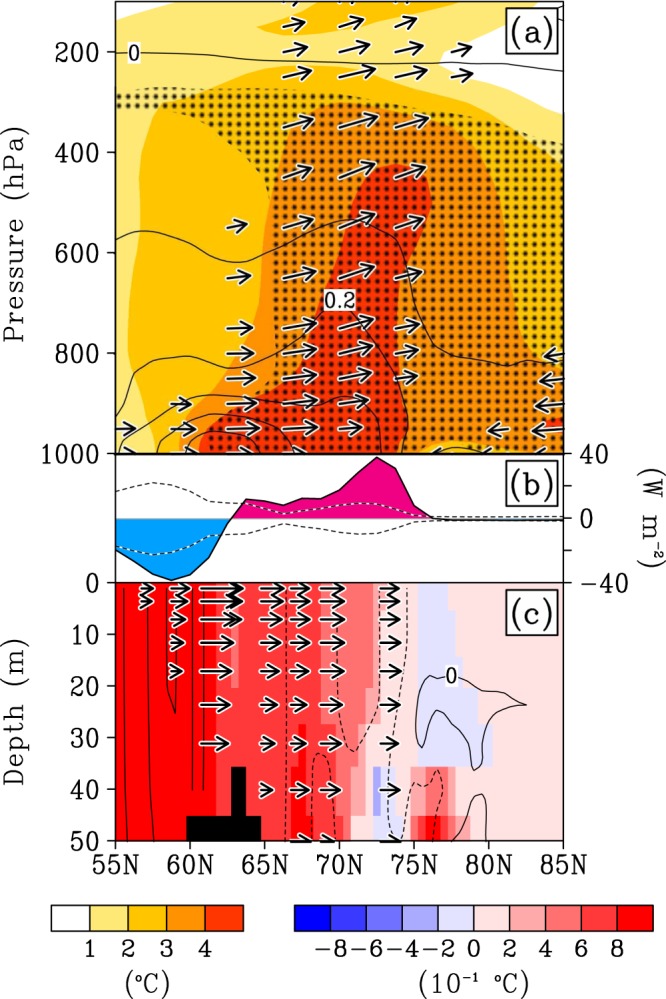
Vertical-meridional section showing longitudinally averaged atmospheric and oceanic fields covering Bering-Chukchi region in winter 2017–18. (**a**) Three-month mean atmospheric fields averaged from 160°E to 210°E in winter 2017–18. Color: warm air temperature anomaly; Arrow: northward and upward wind anomaly; Contour: positive specific humidity anomaly. Contours of positive specific humidity anomaly are shown at an interval of 0.1 g kg^−1^. Hatching areas show that the temperature anomaly exceeded two times as large as one standard deviation. (**b**) The sum of latent and sensible heat flux anomaly, upward direction defines positive (red), and downward is negative (blue). The width of one standard deviation was drawn by broken lines. (**c**) Three-month mean oceanic fields along 191°E in winter 2017–18. Black-boxed area corresponds to the region of the Bering Strait. Shade: ocean temperature anomaly. Arrow: time change of northward ocean current from 12 November to 15 February in 2017–18 winter, minus climatological time change. Contour: time change of temperature from 12 November to 15 February in winter 2017–18, minus climatological time change. Solid line indicates time change of ocean temperature is positive—i.e., warming; whereas broken line is negative—i.e., cooling. Contour interval is 0.01 °C day^−1^.

Next we show a vertical-meridional section of air temperature, specific humidity and northward and upward wind covering the Bering-Chukchi region in winter 2017–18 (Fig. 6a). The moisture anomaly propagated poleward and upward over the area of the warm hole. This poleward and upward flow made the upper Arctic air warm and humid, because the upward flow overlying sea ice provides the upper atmosphere with extra heat via condensation of water vapour^13^. In addition, anomalous upward heat flux from the area of the warm hole to the atmosphere assisted atmospheric warming (Fig. 6b). The air mass over the sea-ice hole is warmer than the air mass over the surrounding ice-covered areas. This assists the transportation of warm air to upper levels. These processes form a boot-shaped atmospheric heat profile, shown by a reddish color area in Fig. 6a. The Bering Strait warm hole is therefore responsible for propagation of the warm air into the Arctic^21^. The formation of the upper-level warm air is equivalent to upper air high pressure, due to the hydrostatic relation.

### Discussion of possible positive feedback process

This section interprets how the warm hole influences the jet meander responsible for the cold mid-latitudes. Poleward intrusion of moist air strengthens an accompanying cyclone^13,15,22^ owing to latent heat release by cloud formation. The strengthened cyclone further strengthens the poleward intrusion of moist air^13^. Thus, the cyclone centred to the west of the Bering Sea shown in Fig. 5b likely strengthened the poleward moisture flux, which was then able to pass through the Bering Strait to the Chukchi Sea. Anomalous upward heat flux from the area of the warm hole (Fig. 6b) further strengthened the surface cyclone. Similar thermal forcing of cyclones has commonly been seen over mid-latitude regions where there are large horizontal sea surface temperature (SST) gradients, such as the Kuroshio Current and Gulf Stream^23,24^. Because the thermal gradient due to the sea ice is probably larger than the mid-latitude SST, it is logical to assume that the sea-ice-induced cyclone development is not unusual over the Arctic region. In fact, a numerical experiment for a polar low, which appeared over the Barents Sea near a sea-ice edge, showed that a more intense low develops in warm SSTs than in cold SSTs^25^.

The strengthened cyclone could potentially have an influence on ocean currents. Anomalous northward wind stress in association with the cyclone will allow mid-latitude warm water to penetrate across the Bering Strait into the Chukchi Sea^26^ (Fig. 6c). This will be favourable for maintaining the warm hole, as the northward wind stress will further accelerate northward sea-ice retreat^27^.

The upward heat flux overlying the warm ocean implies that the ocean actively heats the atmosphere (Fig. 6b). In the Bering Sea, in contrast, heat flux was downward—i.e., from atmosphere to ocean. This implies the Bering Sea was heated by the overlying warm atmosphere (solid lines in Fig. 6c). Therefore, heat was transported from the warm air to the underlying Bering Sea, heat stored in the ocean penetrated through the Bering Strait by the wind driven ocean current, and heat was further transported from the warm hole to the overlying atmosphere. Atmospheric direct heat transport was intermittent, because direct heat transport occurs only during south winds. Meanwhile, heat transport stored in the ocean with large inertia is continuous. Thus, warm Arctic air was possibly sustainable.

Downward infrared radiation from the warm upper air to the ocean surface opposes ice formation^14,16,28^. The winter-mean anomaly of the downward infrared radiation flux at the surface averaged from 160°E to 210°E and from 55°N to 85°N in the 2017–18 winter was larger than 20 W m^−2^, which was approximately equivalent to upward sensible and latent heat fluxes. Upward sensible and latent heat fluxes from an ice-free ocean generally accelerates the ice formation as a result of the ocean losing heat. The anomalous downward infrared radiation, in contrast, contributes to sea-ice melt and delayed freeze-up. This extra downward infrared radiation was caused by warmer and more moist upper air. The delay in closing of the ice-free ocean and warm and humid upper air may have a thermodynamic positive feedback process. Both the thermodynamic process and mechanical northward advection of sea ice due to the northward wind stress could be responsible for maintaining the low ice condition. These atmosphere-ice-ocean feedback processes likely prompted upper-air warming, which was responsible for the jet meander.

We next interpret the anomalous southward jet meander over Asia with consideration for the results of the numerical experiments. As shown in the line of 5300 m in Fig. 2, a portion of the jet from East Asia detoured southward, avoiding the area of the sea-ice hole. The jet split northward and southward as a result. In association with this split, wind speed, which is proportional to the density of the contour, was anomalously weak there. An atmospheric wave theory says that environmental easterly or weak westerly wind conditions are unfavourable for large-scale atmospheric wave propagation^3,4,20,29^. A west-to-east wave from Europe (bluish area), central Siberia (reddish area) and East Asia (bluish area) seen in Fig. 1b was not able to propagate further north-eastward, owing to this environmental weak wind. The blockage of the wave from Europe likely amplifies the wave over East Asia, owing to the dissipation of the wave energy^5^. We speculate that the wave from Europe was partially enhanced by the retreat of Barents Sea ice, as pointed out by many studies^7,9,12^, and that this Eurasian wave was also a necessary condition for the extreme winter. The Barents sea-ice anomaly in the winter worked on a more or less permanent basis for about ten years, with repeatedly cold winters in Eurasia. This year was very unusual; the exceptionally strong anomaly on the other side of the Arctic (i.e., the Chukchi Sea) may have ‘reinforced’ the effect of the ‘semi-permanent’ anomaly in the Barents region. Some resonant effects may have occurred as well. Atmospheric numerical experiments under an ice-free condition in the Chukchi area successfully simulated a cold East Asia and North America (Fig. 3a). The comparison of this experiment with ones under an ice-free condition in the Barents or Greenland-Hudson Bay areas strongly supports the ‘reinforced’ effect of the sea-ice hole. Further specialized results are in the Supplemental Results section.

**Figure 7:**
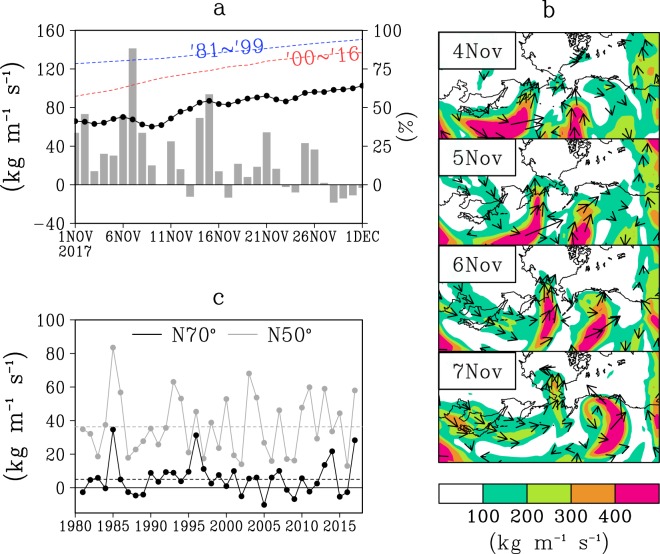
Pacific atmospheric rivers and their intrusion across Bering Strait in the beginning of winter of 2017. (**a**) Black curve shows the evolution of sea-ice concentration in the Pacific side of the Arctic in 2017. The unit is %. Red and blue curves show those of 1981–1999 average, and 2000–2016 average respectively. Bar shows the value of northward moisture flux across 70°N. (**b**) An atmospheric river event shown by vertical integrated vapour flux (IVT) from 4 to 7 November 2017. (**c**) Interannual variation of northward IVT crossing at 50°N and 70°N and their climatological mean values.

We further focus on the beginning of the cold season, from which the positive feedback system may be initiated. Figure 7a shows the daily strength of northward vapour flux latitudinally across the Bering Strait from 1 to 30 November, 2017, and the evolution of the sea ice coverage over the Chukchi Sea. Large water-vapour flux events occurred about four times. The occurrence of the large flux is intermittent, and the strongest event occurred from 5 to 7 November. The strongest event resembles a river (Fig. 7b), in which large values of vertical integrated vapour flux (IVT)^30^ – a measure of an atmospheric river – move from the western North Pacific through the Bering Sea, and are finally pushed into the Chukchi Sea.

Just after this intrusion, sea-ice concentration declined (Fig. 7a). Other sea-ice decline events on 2, 16, and 22 November also occurred just after the large flux events. This implies the influence of the atmospheric rivers upon the maintenance of the warm hole. In fact, the monthly-scale evolution of the sea ice is more gradual than those of recent years (see the slope of red line in Fig. 7a). Additionally, the sea ice in the beginning of November 2017 was much lower than normal (see blue and red lines in Fig. 7a). Northward vapour flux across the latitudes over the Aleutian Archipelago in November 2017 was larger than the climatological norm, but was not exceptionally large (grey line in Fig. 7c). In contrast, the flux across 70°N, i.e., the Bering Strait, was one of the top three values in 2017. Therefore, the anomalous occurrence of atmospheric rivers in the beginning of winter was likely to play a role in anomalous poleward moisture flux, shown in Fig. 5, and the atmospheric rivers prevented the warm hole from closing off.

**Figure 8:**
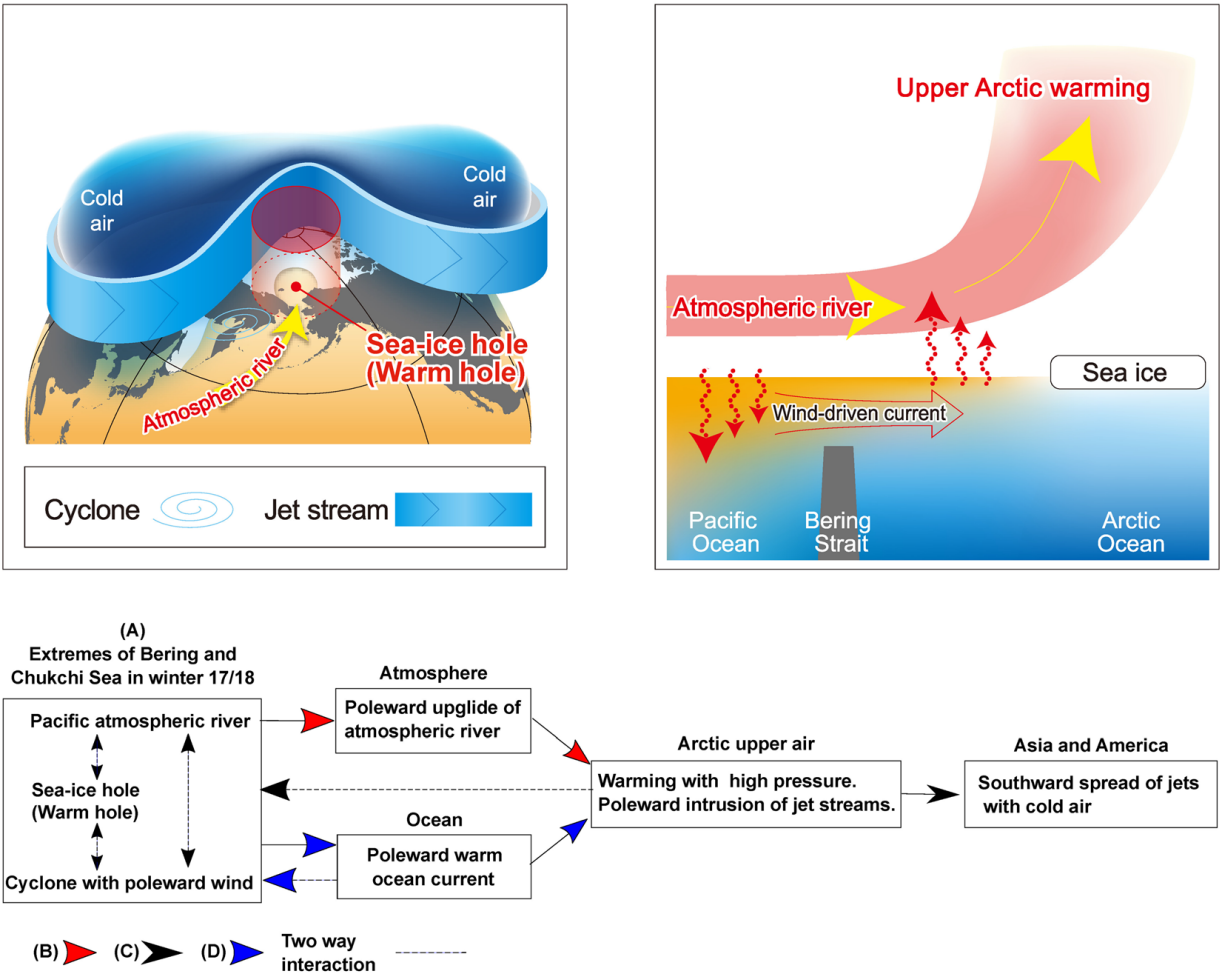
Possible processes for cold Asia and America viewed from an atmosphere-ocean coupled system. (Upper left) Horizontal view of the process that warm Arctic upper air due to an underlying sea-ice hole allowed the jet stream to meander poleward. In response, the jet meandered southward, with cold intrusion into Asia and America. The presence of the warm sea-ice hole resulted in formation of cyclone-derived atmospheric rivers that penetrated into the Arctic with anomalous south winds toward the sea-ice hole. (Upper right) Vertical-meridional view from the Pacific to the Arctic. The injection of atmospheric rivers due to anomalous south winds made upper Arctic air warm, leading to poleward upward wind. The wind-driven ocean current injected warm seawater into the sea-ice hole, which further heated the overlying air. (Bottom) Symbols, (A–D), in the flow chart respectively correspond to individual processes. The process (A) indicates positive feedback among the atmospheric river, warm hole and cyclone. Red arrows (B) show local atmospheric processes that made upper air warm, illustrating in the upper right panel. Black arrows (C) show large-scale atmospheric processes, illustrating in the upper left panel. Blue arrows (D) show local oceanic processes illustrated in the upper right panel. Dotted thin arrows show feedback processes.

The summaries of the overall processes that caused East Asia and North America to be cold are illustrated in Fig. 8. Based on Figs 2a and 7a,c, it appears that 2017 was the first year when anomalously strong early winter atmospheric-river activity in the Chukchi Sea interacted with pre-existing low sea ice concentration. This interaction between pre-existing low sea ice concentration and anomalous atmospheric-river activity helped to maintain the warm hole. We demonstrate that a leading factor determining the interannual variation of atmospheric rivers is not the warm hole alone. Rather, the warm hole acted in concert with the poleward intrusion of the atmospheric river to initiate a self-enhancing process: magnifying the intrusion effect in a northward jet meander (Fig. 8).

## Conclusion

We showed that a warm hole over the Pacific sector was responsible for the cold 2017–18 winter in the mid-latitudes, in addition to the effect of the semi-permanent ice retreat in the Barents Sea. The warm hole symbolizes the historically largest ice-free ocean in the Bering and Chukchi Seas because the shape of the ice-free ocean appears as a hole with overlying warm air. Analyses focusing on the area of the Pacific sector also showed that there was a positive feedback mechanism between the warm hole, underlying ocean, and overlying atmosphere. This feedback process made the Arctic upper air warm, which caused the jet stream to push northward. Furthermore, we showed that the coincidence of the two events – pre-existing warm holes in late autumn and the occurrence of the sporadic strong and moist northward wind events that are strong enough to move thin ice, such as an atmospheric river, – could initiate and develop the positive feedback system. In addition, the poleward penetration of atmospheric rivers can induce sea ice melt or prevent its formation through enhanced downwelling longwave radiation. The positive feedback between the large ice-free ocean, the atmospheric rivers, and the ocean current actively pushed the jet northward. In reaction to this northward meander, large southward jet stream pathways formed over Asia and America, allowing cold air to spread into Asia and the southern areas of North America. We therefore conclude that the warm hole apparing in the Pacific side of the Arctic is responsible for the anomalous jet meander. The positive feedback system does not operate in isolation within the Arctic. Rather there are contributions from external lower-latitude forcings, such as atmospheric rivers^16^. Deepening the knowledge of the extra-Arctic processes that control long-term variations of atmospheric rivers is beneficial for long-range winter weather forecasts. Examining the reasons why the warm hole appeared is also beneficial because the sea-ice hole serves as a predictor. The positive feedback could provide Eastern Eurasia and North America with cold winters in the new era of the warm hole. Whether the positive feedback is strong or not must be tested by examining detailed data analyses, together with high-resolution numerical simulations of both the atmosphere and ocean. This study showed an upscale effect, i.e., influence of narrow-width atmospheric rivers upon global-scale jet streams, suggesting that a fine-mesh numerical model that resolves atmospheric rivers is needed to predict the global-scale climate change associated with future global warming.

## Methods

### Atmospheric data

We used the gridded atmospheric reanalysis dataset of the Japanese 55-year Reanalysis (JRA-55), supplied by the Japanese Meteorological Agency^31^. The definition of anomaly is the deviation from long-term mean values from 1980–81 through winter 2017–18. To overview hemispheric scale atmospheric fields, the index of seasonal variations of the Northern Hemisphere annular mode (SV NAM)^32^ was used in this study. The SV NAM, a leading mode of EOF analysis applying vertically and zonally averaged atmospheric geopotential height fields poleward of 40°N, is able to identify the overall atmospheric pressure contrast between the Arctic region and the mid-latitude region better than the well-known AO index. In addition, SV NAM is a good indicator for the strength of the polar jet stream. The SV NAM index is available at http://www.bio.mie-u.ac.jp/kankyo/shizen/lab1/AOindex.htm. Figure S2 shows time series of SV NAM index from August of 2017 through February 2018. The index was negative overall from the middle of November to the middle of February, except for a short discontinuation in the middle of December. No previous winters experienced such a long-lasting negative phase of SV NAM as in the 2017–18 winter. Although some winters’ monthly mean indices were less than in this particular winter (as in 2010), even in these years the negative phase did not continue for three months. We therefore focus on a three-month period between 15 November 2017 to 15 February 2018. Thus, most atmospheric and oceanic maps shown without notice otherwise are the time-mean for this period. We have confirmed that the choice of the period did not largely change the pattern of figures. The present study mainly focuses on atmospheric anomalous fields in the winter of 2017–18 as compared with long-year-mean climatology. To statistically show their anomalousness in 2017–18, standard deviations calculated from inter-annual variation are also superimposed in the figures.

Arrows in Fig. 5a indicate that the horizontal moisture flux multiplied by the specific humidity of the zonal and meridional wind components is integrated from 1000 hPa to 300 hPa. The absolute value of the arrow is usually referred to as IVT, the calculation of which is used widely for detecting atmospheric rivers^30^. Atmospheric rivers are detected by identifying contiguous regions ≥2000 km in length with IVTs ≥ 250 kg m^−1^ s^−1^ in mid-latitude regions^29^, and IVTs ≥ 200 kg m^−1^ s^−1^, IVTs ≥ 150 kg m^−1^ s^−1^, or IVTs ≥ 100 kg m^−1^ s^−1^ for high-latitude regions^13,33,34^. Figure 7 shows the calculated IVT and its northward component. River-style high IVT value areas entering from the Pacific through the Bering Strait, and entering into the Arctic shown in Fig. 7b satisfy the aforementioned criterion for the atmospheric river. The northward components across 50°N and 70°N from 160°E to 150°W are calculated, respectively, as well. The former represents the strength of moisture flux from the Pacific Ocean into the Bering Sea, and the latter is the flux entering the Arctic Ocean from the Bering Sea.

### Ocean and sea ice data

For sea ice data, we used ERA-Interim^35^, supplied by the European Centre for Medium-Range Weather Forecasts from 1981 through 2018. The sea ice concentration shown in Fig. 2 is monthly averaged sea ice concentration in November 2017. The ice concentration deviation of the 2017–18 winter from those of the recent five-year mean is shown in Fig. 2b. In the recent years, sea ice has shown a retreating trend. Comparison with the most recent five-year mean shows the anomalousness of this 2017–18 year. Day-by-day evolution of sea-ice concentration in the Pacific side of the Arctic in 2017 in association with day-by-day fluctuation of atmospheric rivers is shown in Fig. 7. Because ERA-Interim is an assimilation product in which numerically simulated values are reflected in the areas of no observation, ERA-Interim is advantageous for seeing the day-by-day interaction between the atmosphere and sea-ice. As for the monthly mean ice concentration seen in Fig. 2, the difference from other observational product is small.

The ocean reanalysis product used in the present report is the Multivariate Ocean Variational Estimation system/Meteorological Research Institute Community Ocean Model (MOVE/MRI-G2)^36^. Its horizontal resolution is 1 × 1 degrees with 50 vertical layers. Time resolution for this product is every ten days. The definition of the anomaly is the deviation from long-term mean values from 1981 through 2010. Figure 6c demonstrates this product. The averaged period for a winter is from 12 November 2017 through 15 February. The surface heat flux shown in Fig. 6b is from the JRA-55 reanalysis dataset. Arrows shown in the lower panel represent the time change of the ocean current on 15 February 2018 from 12 November 2017, minus the climatological time change. The time change reflects the influence of wind stress. The black-colored box area, corresponding to the Bering Strait, indicates the bottom of the ocean. The bottom panel shows meridional-vertical oceanic fields along the meridional line of 191°E to demonstrate oceanic character across the Bering Strait. Over the Bering Strait, northward anomalies are seen in particular in the surface. In order to clarify air-sea interaction, we vertically arranged vertical-meridional cross sections of the atmosphere and ocean, and the sum of latent and sensible heat flux. Atmospheric fields shown in Fig. 6 are the longitudinal average from 160°E through 210°E. These atmospheric-oceanic vertical-meridional combined panels over Bering-Arctic areas are able to demonstrate the influence of the wind over the ocean currents, and the influence of the ocean temperature over the atmospheric thermodynamics.

### Numerical experiment

We employed Weather Research and Forecasting (WRF) version 3.9.1^37^ for this experiment, and the simulation area covers most of the area to the North of 10°N. The simulation domain is gridded into 150 × 150 points, with a horizontal grid spacing of 100 km. There are thirty atmospheric layers vertically, with the top of atmosphere set to 10 hPa. There are five ensemble members for each group of sensitivity tests; the first ensemble member starts on 1 August 2017, and ends on 30 April 2018. The other four members start respectively on 30 July, 31 Jul, 2 August, and 3 August, and all end on the same date as the first member.

NCEP/NCAR reanalysis (NNRP)^38^ data is employed as the forcing, and provide atmospheric background as lateral boundary conditions and surface cover, including SST/sea ice conditions for surface boundary condition. Three zones of interest are identified, for which the original sea ice cover (2017–2018) is replaced with sea ice values from 1983–1984 for sensitivity simulations for each ensemble member. Zone 1 is delimited by 135°E and 135°W longitude, containing the oceanic region of the East Siberia Sea, the Beaufort Sea, and the Chukchi Sea. Zone 2 is delimited by 45°W and 90°E, containing the North Atlantic Ocean, the Barents Sea, and the Kara Sea. The third zone has the boundaries of 135°W and 45°W in longitude, which basically covers the oceanic area in the North of Canada, as well as to the West of Greenland.

All runs included in the analysis are forced by the 2017–2018 atmospheric boundary conditions (from NNRP). Low sea ice runs are forced by 2017–2018 sea ice conditions, and high sea ice runs are forced by the 1983–1984 sea ice conditions. The sea ice conditions are also from NNRP. The anomalies are calculated by subtracting variables (e.g., temperature and geopotential height) of reference high sea ice cases from low sea ice cases. The sea ice conditions are imported into WRF as 6-hourly sea ice extent at the surface boundary. Thus the sea ice extent changes through time rather than being fixed.

## Supplementary information

SUPPLEMENTARY INFORMATION

## Data Availability

The analysed datasets along with the initial and boundary conditions of the numerical experiments are open to the public as indicated in the Method section.
