# Author Correction: Warm hole in Pacific Arctic sea ice cover forced mid-latitude Northern Hemisphere cooling during winter 2017–18

**DOI:** 10.1038/s41598-020-62000-3

**Published:** 2020-03-17

**Authors:** Yoshihiro Tachibana, Kensuke K. Komatsu, Vladimir A. Alexeev, Lei Cai, Yuta Ando

**Affiliations:** 1 Faculty of Bioresources, Mie University, Tsu, Japan; 2 International Arctic Research Center, University of Alaska Fairbanks, Fairbanks, USA

Correction to: *Scientific Reports* 10.1038/s41598-019-41682-4, published online 03 April 2019

This Article contains typographical errors in the Acknowledgements section.

“Y.T., K.K.K. and Y.A. were partly supported by the Ministry of Education, Culture, Sports, Science and Technology through a Grant-in-Aid for Scientific Research on Innovative Areas (Grant Number 16K13880 and 17H02958), Arctic Challenge for Sustainability Project, Belmont Forum InterDec Project, National Institute of Polar Research (NIPR) through General Collaboration Project No. 29-25, and the collaborative research program (29G-01) of the Disaster Prevention Research Institute of Kyoto University. V.A. and L.C. was partially supported by NSF Grants ARC-1417300, 1203473, and National Oceanic and Atmospheric Administration cooperative agreement NA13OAR4320056 with the University of Alaska. The authors thank Dr. Kaja Brix for reading the paper and providing valuable comments.”

should read:

“Y.T, K.K.K and Y.A. were partly supported by the Ministry of Education, Culture, Sports, Science and Technology through a Grant-in-Aid for Scientific Research on Innovative Areas (Grant Number 16K13880 and 17H02958), Arctic Challenge for Sustainability Project, Belmont Forum InterDec Project, National Institute of Polar Research (NIPR) through General Collaboration Project No. 29-25, and the collaborative research program (29G-01) of the Disaster Prevention Research Institute of Kyoto University. V.A and L.C were partially supported by Alaska EPSCOR, and National Oceanic and Atmospheric Administration cooperative agreement NA13OAR4320056 with the University of Alaska. The authors thank Dr. Kaja Brix for reading the paper and providing valuable comments.”

In addition, the legend of Figure 3 is incorrect:

“Atmospheric response under the 2017–18 sea-ice boundary condition by a numerical simulation in the lower troposphere. (**a**) Atmospheric deviation fields in February when sea-ice boundary condition over a specific region is set in 2017–18 from those of 1983–84. (**a**) Bering-Chukchi region, (**b**) Barents-Kara region, (**c**) Greenland-Hudson Bay region, (**d**) the sea ice of all the three regions is set in 2017–18.”

should read:

“Atmospheric response under the 2017–18 sea-ice boundary condition by a numerical simulation in the lower troposphere. Atmospheric deviation fields in February when sea-ice boundary condition over a specific region is set in 2017–18 from those of 1983–84. (**a**) Bering-Chukchi region, (**b**) Barents-Kara region, (**c**) Greenland-Hudson Bay region, (**d**) the sea ice of all the three regions is set in 2017–18.”

Finally, the legend of Figure 4 is incorrect:

“Atmospheric response under the 2017–18 sea-ice boundary condition by a numerical simulation in the middle troposphere. (**a**) Atmospheric deviation fields in February when sea-ice boundary condition over a specific region is set in 2017–18 from those of 1983–84. (**a**) Bering-Chukchi region, (**b**) Barents-Kara region, (**c**) Greenland-Hudson Bay region, (**d**) the sea ice of all the three regions is set in 2017–18.”

should read:

“Atmospheric response under the 2017–18 sea-ice boundary condition by a numerical simulation in the middle troposphere. Atmospheric deviation fields in February when sea-ice boundary condition over a specific region is set in 2017–18 from those of 1983–84. (**a**) Bering-Chukchi region, (**b**) Barents-Kara region, (**c**) Greenland-Hudson Bay region, (**d**) the sea ice of all the three regions is set in 2017–18.”

