# Supplementary material for: Warm hole in Pacific Arctic sea ice cover forced mid-latitude Northern Hemisphere cooling during winter 2017–18
Source: Sci Rep. 2019 Apr 3;9:5567. doi: 10.1038/s41598-019-41682-4 (PMC6447575; doi:10.1038/s41598-019-41682-4)
Supplement: Supplementary file 1 — SUPPLEMENTARY INFORMATION [file 41598_2019_41682_MOESM1_ESM.pdf]

SUPPLEMENTARY INFORMATION

Warm hole in Pacific Arctic sea ice cover forced mid-latitude Northern Hemisphere cooling during winter 2017-18

Yoshihiro Tachibana[1*], Kensuke K. Komatsu[1], Vladimir A. Alexeev[2], Lei Cai[2] and Yuta Ando[1]

[1]Faculty of Bioresources, Mie University, Tsu, Japan

[2]International Arctic Research Center, University of Alaska Fairbanks, Fairbanks, USA

***Corresponding author:** Yoshihiro Tachibana

E-mail: tachi@bio.mie-u.ac.jp

Discussion on the meander of jet(s)

In the main text, we have not described the southward meander in detail. So here we focus particularly on the meander over Asia, with theoretical consideration and numerical experiments. As in the main text, a portion of the jet from East Asia detoured southward, avoiding the sea-ice hole. This jet had split northward and southward, avoiding the Bering and Chukchi region. In association with this split, wind speed was anomalously weak there. The region of the weak wind spread westward. In association with the negative westerly anomaly region from about 50°N to 85°N, where the weak or easterly area is dominant. Owing to these weak westerlies, a west-to-east wave from Europe (as seen in Fig. 1b and 1c) is expected to be unable to propagate further eastward. The degree of the northward gradient of potential vorticity is a good measure of the suitability of large-scale atmospheric wave propagation. Fig. S3 shows longitudinal average potential vorticity in the meridional-vertical cross section. Potential temperature is used as the vertical axis. The potential vorticity gradient northward from 45°N was almost flat and associated with negative potential vorticity. This indicates the air mass was stagnant and without wave propagation there. Because potential temperature and potential vorticity conserve their values in the adiabatic and frictionless conditions, the atmosphere is freely able to move northward or southward within an isentrope and in the area of flat potential vorticity. Thus, the blockage of the wave from Europe likely amplifies the wave over East Asia, owing to the dissipation of the wave energy[1] In addition, the flat potential vorticity gradient allows the air to freely move northward or southward, presenting favorable conditions for an isolated vortex such as a cut-off low or a blocking high[2, 3-5].

Next we show the results of a simple linear baroclinic model (LBM)[6], in which the response to a given heating from climatological mean fields is calculated. LBM is a useful tool for diagnosing the primary response of the atmosphere without secondary feedback. Referring to the vertical structure of the temperature anomaly shown in Fig. 6, the heat source is given as in Fig. S4 over the Bering and Chukchi and Barents regions. Fig. S5 shows the responses of geopotential height and temperature to the given heat. The response to the forcing of the Chukchi Sea shows cold East Asia and warm Chukchi Sea regions, with positive 500 hPa height anomalies over the Arctic, and negative high anomalies over the North Pacific. The results are in agreement with the observed anomalies shown in Fig. 1b, with little eastward phase shift. The response patterns also in agreement with Asian–Bering–North American teleconnection[7] and another simple numerical model[8]. The response to the forcing of the Barents Sea also shows a cold East Asia, though the horizontal 500 hPa anomaly patterns less resemble observations than a response to the Chukchi Sea.

Consider again the WRF simulation shown in Figs. 3 and 4. As described in the main text, a cold Northeast Asia and North America and warm Arctic are successfully simulated as a response to the Chukchi sea-ice hole. Interestingly, northeast Asia and North America are always cool when the ice-free region is over the Barents or Greenland-Hudson Bay as well the Chukchi Sea. It should be noted that when all three regions are ice-free—which is the condition of this winter—the atmospheric response pattern is more amplified than those of the simple sum of each run.

From these results, we can arrive at one following possible interpretation. The Barents Sea ice and temperature anomaly in the winter has been working on a more or less permanent basis for about ten years, with repeatedly cold winters in Eurasia, noted by many studies. This

year was quite unusual—the exceptionally strong anomaly on the other (Chukchi Sea) side of the Arctic has 'reinforced' the effect of the 'semi-permanent' anomaly in the Barents region. There could be some resonant effects in play here as well. Each of these anomalies could excite wavenumber 2 in the midlatitudes, with maxima in the same locations. The Arctic is too small on the global scale, so any sizable heating anomaly within the Arctic will cause a similar response. If two anomalies are acting, they can have a reinforcing effect. It seems that this year had another 'helper' in the Chukchi region to make wavenumber 2 very strong.

The southward meander in the North American side is reasonable, as it is located in the downstream side of the northward jet meander. The American side meander is in agreement with a previous numerical simulation study, where an ideal atmospheric numerical simulation without sea ice over the Chukchi Sea region simulated cold weather over North America[9]. A baroclinic atmospheric theory demonstrates that the atmospheric trough-ridge system essentially tends to tilt westward with the increase of height in the environmental upper westerly condition as the system develops. A developing surface cyclone thus tends to be located between a left trough and right ridge[10, 11]. The location of the surface cyclone shown in Fig. 5b was between the upper Asian deep trough and the ridge over Bering Sea. Thus, the surface cyclone might further develop, owing to the jet meander. Because a strengthened cyclone near the Bering Sea is favorable for initiating the system shown in the main text, there might be an additional positive feedback system between a large-scale atmospheric wave and the sea ice hole. This will be left to a future study.

As in previous studies, the influence of sea-ice retreat over a large-scale westerly jet is not easily seen in a single cold wave event, but only manages to be seen by statistical analyses using a long-year dataset or numerical simulations with many samples[12,13], as the variability of

the jet meandering owing to atmospheric internal fluctuation is larger than that owing to surface conditions such as sea ice[14]. In this winter, there were many short-lasting extreme cold wave events with a southward meandering jet. Some of them might not be related to the sea-ice hole, but to atmospheric internal oscillation. Viewing many cold wave events from a seasonal mean might be able to filter out atmospheric internal oscillation, similar to considering an ensemble of many members of a numerical simulation.

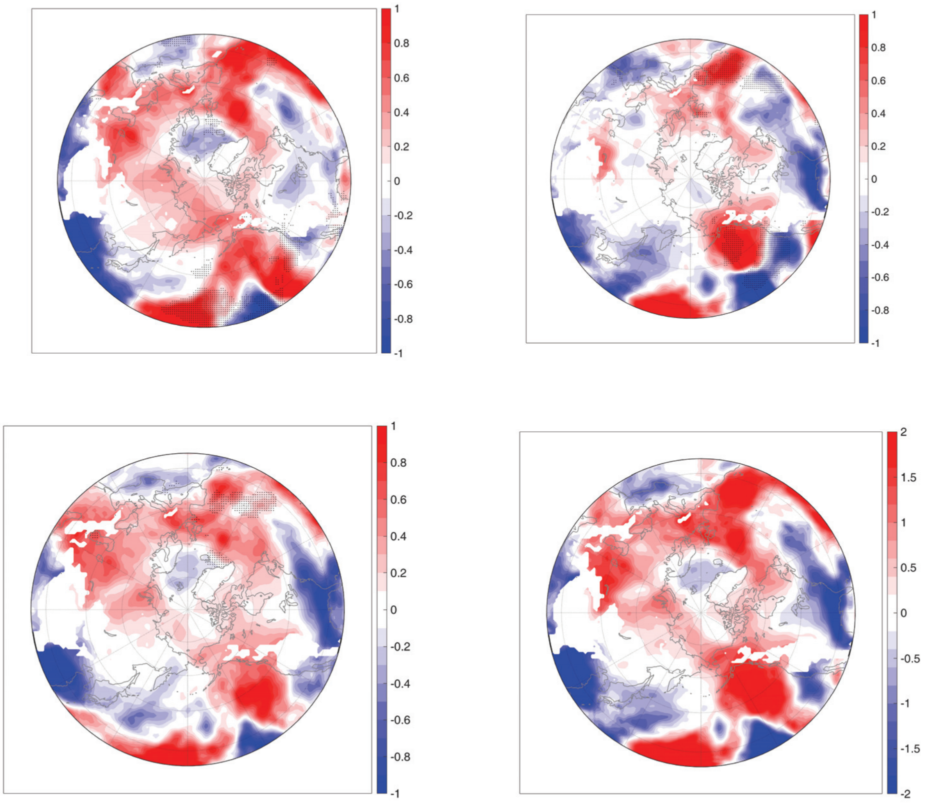

Supplementary Figure 1 Atmospheric response under the 2017/18 sea-ice boundary condition by a numerical simulation. (a) Atmospheric deviation fields in February when sea-ice boundary condition over a specific region is set in 2017/18 from those of 1983/84. (a) Bering-Chukchi region, (b) Barents-Kara region, (c) Greenland-Hudson Bay region, (d) the sea ice of all the three regions is set in 2017/18. Shaded are 850hP specific humidity deviations. The unit is g kg^{-1}. Hatching areas indicate the humidity deviation exceeds 95% confidence level by *t*-test.

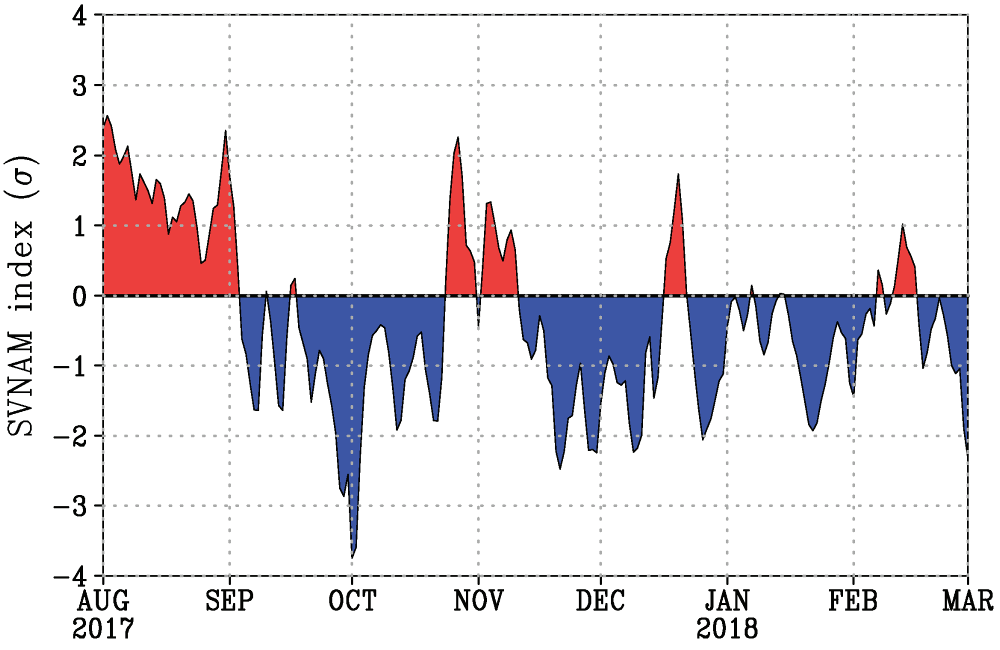

Supplementary Figure 2. SV NAM (AO) index from August to February 2017/2018. The SV NAM index is available at http://www.bio.mie-u.ac.jp/kankyo/shizen/lab1/AOindex.htm.

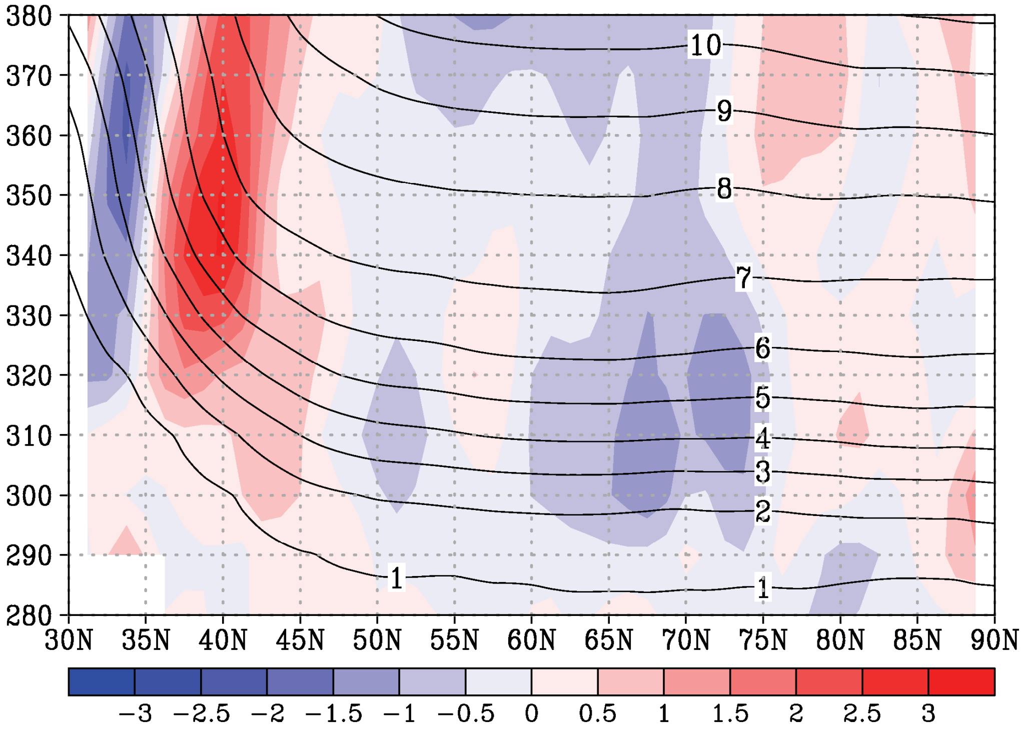

Supplementary Figure 3. Meridional-vertical section of isentropic potential vorticity (contour), and its anomaly from climatological mean (color) (15 November–15 February). Vertical axis indicates the height measured by potential temperature. The values are averaged from 90°E to 180°E. The value is divided by 10^{-6} for potential vorticity (K m^2 kg^{-1} s^{-1}), and multiplied by 10^{-7} for its anomaly (K m^2 kg^{-1} s^{-1}).

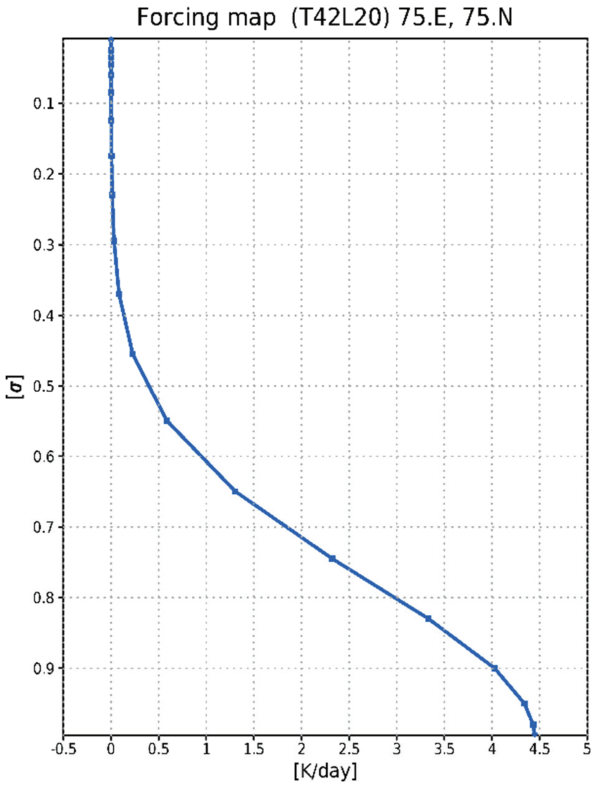

Supplementary Figure 4. Given forcing vertical profile in Linear Baroclinic Model (LVM).

Vertical axis indicates the height as sigma coordinate, and horizontal axis shows given heat.

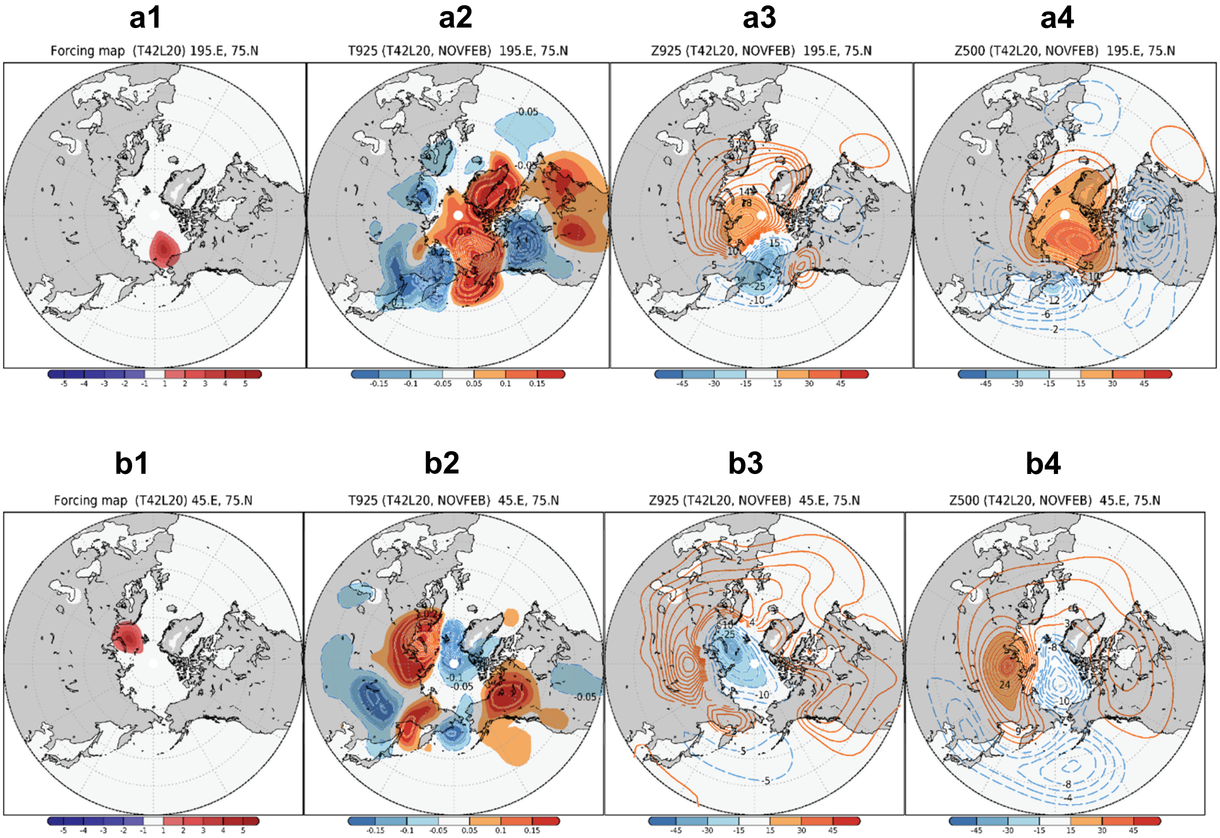

Supplementary Figure 5. Atmospheric response to given forcing. Red color of a1 and b1 indicates forcing area. (a2, b2) Response of 925 hPa temperature from (a1) and (b1) forcing, respectively (K). (a3, b3) As in (a2, b2) but for 925 hPa height (m). (a4, b4) As in (a2, b2), but for 500 hPa height.

References

1. Nakamura, H. & Fukamachi, T. Evolution and dynamics of summertime blocking over the Far East and the associated surface Okhotsk high. *Quart. J. R. Meteorol. Soc.* **130**, 1213–1233 (2004).

2. Cattiaux, J., Peings, Y., Saint-Martin, D., Trou-Kechout, N. & Vavrus, S. J. Sinuosity of midlatitude atmospheric flow in a warming world. *Geophys. Res. Lett.* **43**, 8259–8268 doi:10.1002/2016GL070309 (2016).

3. Francis, J. A. & Vavrus, S. J. Evidence linking Arctic amplification to extreme weather in mid-latitudes. *Geophys. Res. Lett.* **39**, L06801 doi:10.1029/2012GL051000 (2012).

4. Maeda, S., Kobayashi, C., Takano, K. & Tsuyuki, T. Relationship between singular modes of blocking flow and high-frequency eddies. *J. Meteorol. Soc. Japan* **78**, 631– 646 (2000).

5. Tachibana, Y., Nakamura, T., Komiya, H. & Takahashi, M., Abrupt evolution of the summer Northern Hemisphere annular mode and its association with blocking. *J. Geophys. Res.* **115**, D12125 doi:10.1029/2009JD012894 (2010).

6. Watanabe, M. & Kimoto, M. Atmosphere-ocean thermal coupling in the Northern Atlantic: a positive feedback. *Quart. J. R. Meteorol. Soc.* **126**, 3343–3369 (2000).

7. Yu, B., Lin, H., Wu, Z. W. & Merryfield, W. J. The Asian–Bering–North American teleconnection: seasonality, maintenance, and climate impact on North America. *Clim. Dyn.* **50**, 2023–2038 doi:10.1007/s00382-017-3734-6 (2018).

8. McKenna, C. M., Bracegirdle, T. J., Shuckburgh, E. F., Haynes, P. H., & Joshi, M. M. Arctic sea ice loss in different regions leads to contrasting Northern Hemisphere impacts. *Geophys. Res. Lett.* **45**, 945–954 doi:10.1002/2017GL076433 (2018).

9. Screen, J. A. Simulated atmospheric response to regional and pan-Arctic sea-ice loss. *J. Clim.* **30,** 3945–3962 doi:10.1175/JCLI-D-16-0197.1 (2017).

10. Alexander, M. A., Bhatt, U. S., Walsh, J. E., Timlin, M. S., Miller, J. S., & Scott, J. D. The atmospheric response to realistic Arctic sea ice anomalies in an AGCM during winter. *J. Clim.* **17**, 890-905 (2004).

11. Honda, M., Yamazaki, K., Tachibana, Y. & Takeuchi, K. Influence of Okhotsk sea-ice extent on atmospheric circulation. *Geophys. Res. Lett.* **23**, 3595-3598 (1996).

12. Mori, M., Watanabe, M., Shiogama H., Inoue J. & Kimoto, M. Robust Arctic sea-ice influence on the frequent Eurasian cold winters in past decades. *Nat. Geosci.* **7**, 869–873 (2014).

13. Nakamura, T., Yamazaki, K., Iwamoto, K., Honda, M., Miyoshi, Y., Ogawa, Y., Tomikawa Y. & Ukita, J. The stratospheric pathway for Arctic impacts on mid-latitude climate. *Geophys. Res. Lett.* **43**, 3494-3501 doi:10.1002/2016GL068330 (2016).

14. Honda, M., Inoue, J. & Yamane, S. Influence of low Arctic sea-ice minima on anomalously cold Eurasian winters. *Geophys. Res. Lett.* **36**, doi:10.1029/2008GL037079 (2009).
